# In the Beginning Was the Bud: Phytochemicals from Olive (*Olea europaea* L.) Vegetative Buds and Their Biological Properties

**DOI:** 10.3390/metabo13020237

**Published:** 2023-02-05

**Authors:** Marijana Popović, Franko Burčul, Maja Veršić Bratinčević, Nikolina Režić Mužinić, Danijela Skroza, Roberta Frleta Matas, Marija Nazlić, Tonka Ninčević Runjić, Maja Jukić Špika, Ana Bego, Valerija Dunkić, Elda Vitanović

**Affiliations:** 1 Department of Applied Science, Institute for Adriatic Crops and Karst Reclamation, Put Duilova 11, 21000 Split, Croatia; 2 Department of Analytical Chemistry, Faculty of Chemistry and Technology, University of Split, Ruđera Boškovića 35, 21000 Split, Croatia; 3 Department of Medical Chemistry and Biochemistry, School of Medicine, University of Split, Šoltanska 2, 21000 Split, Croatia; 4 Department of Food Technology and Biotechnology, Faculty of Chemistry and Technology, University of Split, Ruđera Boškovića 35, 21000 Split, Croatia; 5 Center of Excellence for Science and Technology-Integration of Mediterranean Region (STIM), Faculty of Science, University of Split, 21000 Split, Croatia; 6 Department of Biology, Faculty of Science, University of Split, Ruđera Boškovića 33, 21000 Split, Croatia; 7 Department for Plant Sciences, Institute for Adriatic Crops and Karst Reclamation, Put Duilova 11, 21000 Split, Croatia; 8 Centre of Excellence for Biodiversity and Molecular Plant Breeding (CoE CroP-BioDiv), Svetošimunska Cesta 25, 10000 Zagreb, Croatia

**Keywords:** *Olea europaea* L., vegetative buds, essential oil, bud extract, volatile profile, phenolic profile, biological activity, antioxidant activity, antimicrobial activity, cytotoxic activity

## Abstract

Even though *Olea europaea* L. is one of the most important and well-studied crops in the world, embryonic parts of the plants remain largely understudied. In this study, comprehensive phytochemical profiling of olive vegetative buds of two Croatian cultivars, *Lastovka* and *Oblica*, was performed with an analysis of essential oils and methanol extracts as well as biological activities (antioxidant, antimicrobial, and cytotoxic activities). A total of 113 different volatiles were identified in essential oils with hydrocarbons accounting for up to 60.30% and (*Z*)-3-heptadecene being the most abundant compound. Oleacein, oleuropein, and 3-hydroxytyrosol had the highest concentrations of all phenolics in the bud extracts. Other major compounds belong to the chemical classes of sugars, fatty acids, and triterpenoid acids. Antioxidant, antimicrobial, and cytotoxic activities were determined for both cultivars. Apart from antioxidant activity, essential oils had a weak overall biological effect. The extract from cultivar *Lastovka* showed much better antioxidant activity than both isolates with both methods (with an oxygen radical absorbance capacity value of 1835.42 μM TE/g and DPPH IC_50_ of 0.274 mg/mL), as well as antimicrobial activity with the best results against *Listeria monocytogenes*. The human breast adenocarcinoma MDA-MB-231 cell line showed the best response for cultivar *Lastovka* bud extract (IC_50_ = 150 μg/mL) among three human cancer cell lines tested. These results demonstrate great chemical and biological potential that is hidden in olive buds and the need to increase research in the area of embryonic parts of plants.

## 1. Introduction

The olive tree, *Olea europaea* L., is one of the most important crops in the Mediterranean since ancient times [1]. Different parts of the olive tree, including predominantly leaves but also fruits, oil, seeds, and bark, have been used to treat various conditions for centuries [2]. Traditionally, it was used in different forms to heal inflammation, diabetes, the gut, hypertension, asthma, diarrhea, and several other conditions [3]. Nowadays, a great deal of research is performed, especially for the fruits, leaves, and olive oil, resulting in in vitro and in vivo evidence of antioxidant, anti-inflammatory, immunomodulatory, antimicrobial, antiviral, antihypertensive, anticancer, antihyperglycemic, gastroprotective, and several other biological activities [4,5,6]. Olives, as well as olive oil and extra virgin olive oil (EVOO), which are one of the most important olive products, are rich in fatty acids, triacylglycerols, tocopherols, sterols, and phenolic compounds [4,5,7,8]. Olive leaves are also rich in phenolic compounds as well as in polyalcohols and triterpenoids [9] jointly contributing to the beneficial effect that they exert on human health. Given the number of beneficial components in different parts of the olive tree, it is no surprise that the concept of the Mediterranean diet, based on the regular consumption of EVOO and other olive derivatives (amongst other foods), is universally recognized by medical professionals, given that it provides extended health benefits and a protective dietary pattern for disease prevention and health maintenance [10,11].

Olive leaf extract is widely used in phytotherapy for the treatment of various conditions and is generally safe even at high doses [12]. Leaf extracts have several bioactive compounds (predominantly oleuropein (OLE) and hydroxytyrosol) that show positive effects on the parameters related to diabetes [13,14], lipid regulation, hypertension, and protection of the cardiovascular system [15,16]. Recent studies show the beneficial effect of olive leaf extract on healing herpes simplex virus labialis [17] as well as on improving the clinical status of COVID-19 patients [18]. The fruit extract has also recently been explored in experimental animals, with positive effects on hepatic lipid accumulation, chronic fatigue syndrome, and antioxidant capacity [19,20,21].

There was a popular concept in France in the middle of the 20th century named gemmotherapy, with the belief that using extracts derived from meristematic tissues would be more beneficial for human therapy than adult plant parts, since they contain specific bioactive compounds that are later subjected to metabolic transformations [22]. Although some studies on the health benefits of bud extracts have been conducted [23,24,25], the entire area is largely unexplored. Olive trees and olive derivatives are the subjects of the research area of many laboratories and scientists around the world; however, research on phytochemicals from olive buds as well as their biological properties is rare. Only a few studies have reported the chemical composition of olive buds but were mainly oriented on their phenolic compounds and scarce biological activities [26,27].

The aim of this study was to obtain phytochemical profiles of olive vegetative bud essential oil (EO) and methanol extract from two Croatian olive cultivars (cvs.), *Lastovka* and *Oblica*, as well as to investigate some of their biological properties (antioxidant, antimicrobial, and cytotoxic activities). To the best of our knowledge, this is the first report of the chemical profile of essential oils from olive vegetative buds and their biological activities.

## 2. Materials and Methods

### 2.1. Chemicals

Standards of volatile and phenolic compounds were commercially obtained as follows: (*Z*)-3-hexen-1-ol (Toronto research chemicals Inc., Toronto, CA, USA); linalool, nonanal, phenylethyl alcohol, decanal, eugenol, 1,3,5-trimethoxybenzene, 3-hydroxytyrosol, caffeic acid, vanillin, *trans*-*p*-coumaric acid, *trans*-*o*-coumaric acid, apigenin-7-glucoside, oleuropein, pinoresinol, quercetin, luteolin, apigenin, diosmetin tyrosol, rutin, luteolin-7-glucoside, oleacein, and ligstroside from Sigma-Aldrich (St. Louis, MO, USA); verbascoside (HWI group, Rülzheim, Germany); and oleuroside (Phytolab, Vestenbergsgreuth, Germany). Alkane standard solutions C_8_-C_20_ and C_10_, C_20_-C_40,_ and derivatization reagent N,O-Bis(trimethylsilyl)trifluoroacet-amide (BSTFA) were purchased from Sigma-Aldrich (St. Louis, MO, USA); pentane, diethyl-ether, acetonitrile, and methanol were from VWR (Radnor, PA, USA); and anhydrous sodium sulfate was from Kemika (Zagreb, Croatia).

### 2.2. Plant Material

Olive vegetative buds were sampled in the experimental olive orchard in Kaštel Stari (43°55′ N; 16°35′ E) belonging to the Institute for Adriatic Crops and Karst Reclamation. The buds were collected from two Croatian olive cultivars, *Lastovka* and *Oblica,* in April 2020. Part of the samples was stored at −80 °C until further analysis, while the other part was left to dry at room temperature.

### 2.3. Extraction

#### 2.3.1. Essential Oil Distillation

Fresh buds were air-dried at room temperature for 15 days. Afterward, three replicate samples of dried buds (100 g) were simultaneously hydrodistilled in a Clevenger apparatus for 150 min. Pentane and diethyl-ether (1:3) were used as a trap for the essential oil [28]. After the distillation, essential oil samples were dried over anhydrous sodium sulfate, the solvent was evaporated under a stream of nitrogen, and the samples were stored in dark glass vials at 4 °C until analysis.

#### 2.3.2. Methanolic Extraction

The plant material was freeze-dried (FreeZone 2.5, Labconco, Kansas City, MO, USA) and a sample of the dry buds was ground to a coarse powder using a stainless-steel mill (A 11 Analytical mill, IKA, Staufen, Germany). The modified procedure described by Marinova et al. [29] was used to extract phenols. Briefly, 0.25 g of powdered tissue was extracted with 10 mL of methanol/water (80:20, by volume) for 20 min with an ultrasonic bath (Sonorex Digitec DT 100H, Bandelin, Berlin, Germany). An aliquot was centrifuged for 5 min at 14,000 RPM/21,255 RCF (Beckman Instruments J2-21, Palo Alto, CA, USA).

### 2.4. Methanolic Extract Derivatization

Prepared methanolic extracts (1 mL) were evaporated in a centrifugal evaporator (RC10-22, Jouan, Herblain, France) at room temperature until completely dry. A derivatizing agent (50 μL of BSTFA) was added to the dried extracts for 20 min at 20 °C prior to the analysis [30]. Commercial phenolic standards (1 mg) were also derivatized by the addition of 50 μL of BSTFA derivatizing agent for 20 min at 20 °C.

### 2.5. Instrumentation and Chromatographic Conditions

#### 2.5.1. GC-MS Conditions for Essential Oil Analysis

Essential oils were diluted in hexane (*v*/*v*, 1:1000) and analyzed by gas chromatography-mass spectrometry (GC-MS) with gas chromatograph model 8890 GC (Agilent Inc., Santa Clara, CA, USA), equipped with automatic liquid injector model 7693A and tandem mass spectrometer (MS/MS) model 7000D GC/TQ (Agilent Inc., Santa Clara, CA, USA). The samples were separated on a nonpolar HP-5MS UI column (30 m length, inner diameter of 0.25 mm, and stationary phase layer thickness of 0.25 µm, Agilent Inc., Santa Clara, CA, USA). Helium was used as the carrier gas, and the flow rate was set to 1 mL/min. The inlet temperature was set at 250 °C, and the volume of the injected sample was 1 μL, at a split ratio of 1:50. An initial column temperature of 60 °C was set for the first 3 min and then increased to 246 °C at a rate of 3 °C/min and maintained for 25 min. Mass spectrometer conditions were set as follows: ionization energy of 70 eV, ion source temperature of 230 °C, and a scanning range of 40–350 *m*/*z*. The individual peaks were identified by comparison of their retention indices with the series of *n*-hydrocarbons (C_8_–C_40_), along with computer matching of mass spectra with commercial databases (Wiley 9N08 & NIST 2017) as well as by comparison with literature data [31]. The percentages in Table 1 and Table S1 were calculated as the mean value of component percentages on the HP-5MS UI column. All analyses were performed in triplicate.

#### 2.5.2. GC-MS Conditions for Derivatized Extracts’ Analysis

Derivatized extracts were analyzed by gas chromatography-mass spectrometry (GC-MS) with Shimadzu (Kyoto, Japan) Nexis GC-2030 gas chromatograph coupled with Shimadzu QP2020 NX mass detector. Helium was used as the carrier gas with a flow rate of 2.46 mL/min. The samples were separated on nonpolar column SH-5MS (30 m length, inner diameter of 0.25 mm, and stationary phase layer thickness of 0.25 µm, Shimadzu, Kyoto, Japan). The inlet temperature was set at 280 °C, and the volume of the injected sample was 1 μL, at a split ratio of 1:10. The initial column temperature of 120 °C was set for the first 3 min, increased to 292 °C at a rate of 5 °C/min, then increased to 320 °C at a rate of 30 °C/min, and maintained for 17 min. The measurement was performed with a scanning range of 35–750 *m*/*z* and with an electron impact ionization energy of 70 eV [30,32,33]. The identification of compounds in derivatized extracts was performed by comparing their trimethylsilyl (TMS) derivative mass spectra and GC retention indices relative to series of *n*-hydrocarbons, by computer matching with commercial libraries (Wiley 12 & NIST 2020) and those of derivatized phenolic commercial standards, and by comparison with literature data. Sample extracts were injected and analyzed in triplicate.

#### 2.5.3. HPLC Conditions for Phenolics Determination

The separation, identification, and quantification of 19 standards of phenolic compounds were performed using a Shimadzu Nexera LC-40 HPLC system (Shimadzu, Kyoto, Japan), equipped with a UV-VIS detector. The column used for phenolic separation was a C18 reversed-phase chromatography column (250 mm length, 4.6 mm width, and particle size 5 μm; Phenomenex, Torrance, CA, USA). Sample elution was performed at a flow rate of 1 mL/min and the temperature was set to 35 °C. The mobile phase A was ultra-pure water/85% o-phosphoric acid (*v*/*v* 99.8:0.2), and mobile phase B was methanol/acetonitrile (*v*/*v* 1:1), all HPLC grade. The chromatographic conditions were optimized in our laboratory, with a total run time of 55 min using a gradient elution as follows: initially 4% B; 25 min 20% B; 40 min 50% B; 45 min 40% B; 50 min 0% B; 52 min 4% B; and 55 min 4% B. In order to create calibration curves for the quantification of the tested phenolics, six concentration levels were prepared and injected into HPLC in triplicate using an autosampler. Calibration curves’ ranges were 0.5 mg/L–50 mg/L for tyrosol, caffeic acid, vanilin, trans-p-coumaric acid, rutin, verbascoside, luteolin-7-glucoside, trans-o-coumaric acid, apigenin-7-glucoside, oleuroside, ligstroside, pinoresinol, quercetin, luteolin, apigenin, and diosmetin and 25–250 mg/L for 3-hydroxytyrosol, oleacein, and oleuropein. Olive bud extracts were filtered through 0.45 µm polyvinylidene difluoride (PVDF) membrane filters prior to HPLC analysis. All samples were injected in triplicate in a volume of 10 µL, and the results were expressed as mg/kg of olive bud extract.

### 2.6. Antioxidant Activity

#### 2.6.1. Oxygen Radical Absorbance Capacity Assay (ORAC)

The assay was performed on a Tecan Infinite 200 PRO spectrophotometer (TecanTrading AG, Männedorf, Switzerland), using 96-well black polystyrene microtiter plates (Porvair Sciences, Leatherhead, UK), according to a method described by Nazlić et al. [34]. Each reaction contained 180 µL of fluorescein (1 µM), 70 µL of 2,2′-Azobis (2-methyl-propionamidine) dihydrochloride (AAPH, Acros Organics) (300 mM), and 30 µL of plant extract or reference standard Trolox (6.25–50 µM) (Sigma–Aldrich). All experimental solutions were prepared in a phosphate buffer (0.075 mM, pH 7.0). Essential oils were diluted in acetone with a starting concentration of 31.8 µg/mL for *Lastovka* and 35.42 µg/mL for *Oblica* and then further diluted in phosphate buffer for the experiment by 40× and 80×. The extract was prepared in 70% methanol (1 mg/mL) and was further diluted with phosphate buffer to 40 µg/mL. The measurements were performed in triplicate. The ORAC values were expressed as µmol of Trolox equivalents (TE) per gram of isolate (EOs or phenolic compounds).

#### 2.6.2. Measurement of the DPPH Radical Scavenging Activity

The antioxidant capacity of the extracts was assessed by the DPPH method previously described by Nazlić et al. [34]. The assay was performed on a Tecan Infinite 200 PRO spectrophotometer (Tecan-Trading AG, Männedorf, Switzerland) using 96-well microtiter plates for the reaction of reduction of alcoholic DPPH (2,2-diphenyl-1-picrylhydrazyl) solution (Sigma–Aldrich) in the presence of a hydrogen-donating antioxidant. Plant extracts were prepared as follows: essential oils diluted in 70% acetone with a starting concentration of 35.42 µg/mL for *Oblica* EO and 31.8 µg/mL for *Lastovka* EO and bud extracts diluted in 70% methanol with a starting concentration of 1 mg/mL. The first step was pipetting 100 μL of methanol (Kemika, Zagreb, Croatia) and 200 μL standard and/or sample into each well. Serial dilutions of the standard and samples were prepared (starting with the mentioned concentrations for the samples) by pipetting 100 μL from the first row with a multichannel pipette into the wells in the second row and so on to the last row, where 100 μL of the solution was ejected after mixing. In the first column, in 96-well plates, a blank sample was always added (70% methanol), and in the second column, Trolox standard of 200 μM concentration was added. After the last step of adding 100 µL of a methanolic solution of DPPH (200 µM) to each well, the reaction started, and initial absorbance was measured immediately at 517 nm. After 30 min of incubation, the absorbance was measured again, and the percentage of DPPH inhibition was calculated according to the following formula by Yen and Duh [35]:

% inhibition = ((AC(0) − AA(t))/AC(0)) × 100,

where AC(0) is the absorbance of the control at t = 0 min and AA(t) is the absorbance of the antioxidant at t = 30 min. All measurements were performed in triplicate.

### 2.7. Antimicrobial Activity

Evaporated bud extracts were dissolved in 4% DMSO at a concentration of 16 mg/mL in order to determine the minimal inhibitory concentration (MIC) by microdilution-method experiments. Mueller–Hinton broth (MHB) was added in a 1:1 ratio to the diluted extracts and 100 µL of the mixture was subjected to the first wells of the 96-well microtiter plate. Two-fold dilutions were performed in the following adjacent wells (4–0.06 mg/mL). To prepare the inoculum, bacterial cultures were grown in MHB for 24 h. The inoculum size was prepared according to the growth curves of the bacteria in the log phase (1 × 10^5^ colony-forming units (CFU)/mL)). After the addition of 50 µL of the inoculum into each well, each plate was shaken on a microtiter plate shaker for 1 min at 600 rpm (Plate Shaker-Thermostat PST-60 HL, Biosan, Riga, Latvia). Along with the samples, 4% DMSO used in sample preparation was tested as well as a positive control (50 µL of inoculum and 50 µL of broth media), a negative control (50 µL of broth media and 50 µL of essential oil/extract), and a blank (100 µL of broth media). After 24 h of incubation at 37 °C, 20 µL of the indicator of bacterial metabolic activity, 2-p-iodophenyl-3-p-nitrophenyl-5-phenyl tetrazolium chloride (INT, 2 mg/mL), was added. Plates were then shaken in the plate shaker and incubated for 1 h at 37 °C. MIC values were determined visually as the lowest concentration of the extract at which suppression of bacterial growth by the reduction of INT to red formazan was not recorded [36]. The minimal bactericide concentration (MBC) of olive bud essential oils and extracts was determined as the lowest concentration at which no microbial growth was detected. Briefly, MBC is measured by reculturing 10 uL of broth from the wells in which the MIC was determined and from the wells with higher concentrations of the extract on the Mueller–Hinton agar (MHA) plates [37]. After 24 h of incubation, a reduction in bacterial growth (99.9%) was observed, and the lowest number of bacterial colonies represents the MBC. Essential oils and extracts were tested against foodborne pathogen bacteria including two Gram-negative (Escherichia coli ATCC 25922 and Salmonella enteritidis ATCC 13076) and four Gram-positive (Enterococcus faecalis ATCC 29212, Listeria monocytogenes ATCC 7644, Staphylococcus aureus ATCC 25923, and Bacillus cereus ATCC 14579) strains.

### 2.8. Cytotoxic Activity

In order to determine the cytotoxic activity of olive bud essential oils and extracts, a cell viability assay (3-(4,5-dimethylthiazol-2-yl)-2,5-diphenyltetrazolium bromide, MTT) was performed on three cell lines: human breast adenocarcinoma (MDA-MB-231), human breast metastatic adenocarcinoma (MCF-7), and human ovarian carcinoma (OVCAR-3) cell line (LGC Standards) [38,39]. MDA-MB-231, MCF-7, and OVCAR-3 cell lines were incubated overnight in 96-well plates at a density of 9000 cells/well for MDA-MB-231 and MCF-7 and 6000 cells/well for OVCAR-3 followed by incubation with test extracts at concentrations in the range of 1–200 µg/mL for 24 h, 48 h, and 72 h (in triplicate). Afterward, cells were incubated with 0.5 g MTT/L at 37 °C for 2 h; the medium was removed, and 10% dimethylsulfoxide (DMSO) was added for another 10 min at 37 °C. The indicator of metabolically active cells, formazan, was formed and measured at 570 nm using a microplate reader (BioSan, Riga, Latvia). The half maximal inhibitory concentration (IC_50_) value is a quantitative measure that indicates how much of a particular inhibitory substance is needed to inhibit, in vitro, a given biological process or component by 50%. We performed its calculation with Microsoft Excel 2016 with data normalization by the measurements of untreated controls. To determine the differences between tested concentrations, analysis of variance (one-way ANOVA) was performed using Past 3.X software (version 3.14, University of Oslo, Oslo, Norway), with the significance level at *p* < 0.05.

## 3. Results

As far as authors know, this is the first report of volatile compounds identified in olive bud essential oils by gas chromatography-mass spectrometry (GC-MS). Croatian cultivars *Lastovka* and *Oblica* were thoroughly characterized and the results are presented in Table 1 and Table S1 and Figure S1. A total of 113 volatiles from 18 different compound classes were identified in the EOs, with a total of 92.08% (cv. *Lastovka*) and 88.59% (cv. *Oblica*) identified compounds. The main component of cv. *Lastovka* was (*Z*)-3-heptadecene (16.25%), followed by 1,3,5-trimethoxybenzene (12.60%) and tricosane (7.08%), while in cv. *Oblica,* (*Z*)-3-heptadecene (8.13%) was also the most abundant compound followed by nonacosane (7.93%) and heneicosane (6.02%). The most abundant class of compounds in both cvs. were hydrocarbons: saturated hydrocarbons (33.48% and 35.36%, respectively), followed by unsaturated hydrocarbons (22.71% and 13.14%, respectively) and aromatic hydrocarbons (4.11% and 6.19%, respectively), yielding overall 60.3% of hydrocarbons in cv. *Lastovka* and 54.69% in cv. *Oblica*. Other highly represented compounds belong to the classes of aldehydes (9.69% and 11.29%, respectively), heterocyclic compounds (1.70–3.96%), and alcohols (1.69–3.23%). Other identified compounds belong to chemical classes of ketones, esters, organic acids, phenols, terpenes (mono-, sesqui-, and tri-), terpene alcohols (mono-, sesqui-, and di-), and furans.

To deepen the knowledge of phenolic compounds from olive vegetative bud extract from Croatian domestic cvs. *Lastovka* and *Oblica*, we have analyzed their methanol extracts with high-performance liquid chromatography (Table 2, Figure S2).

**Figure 1:**
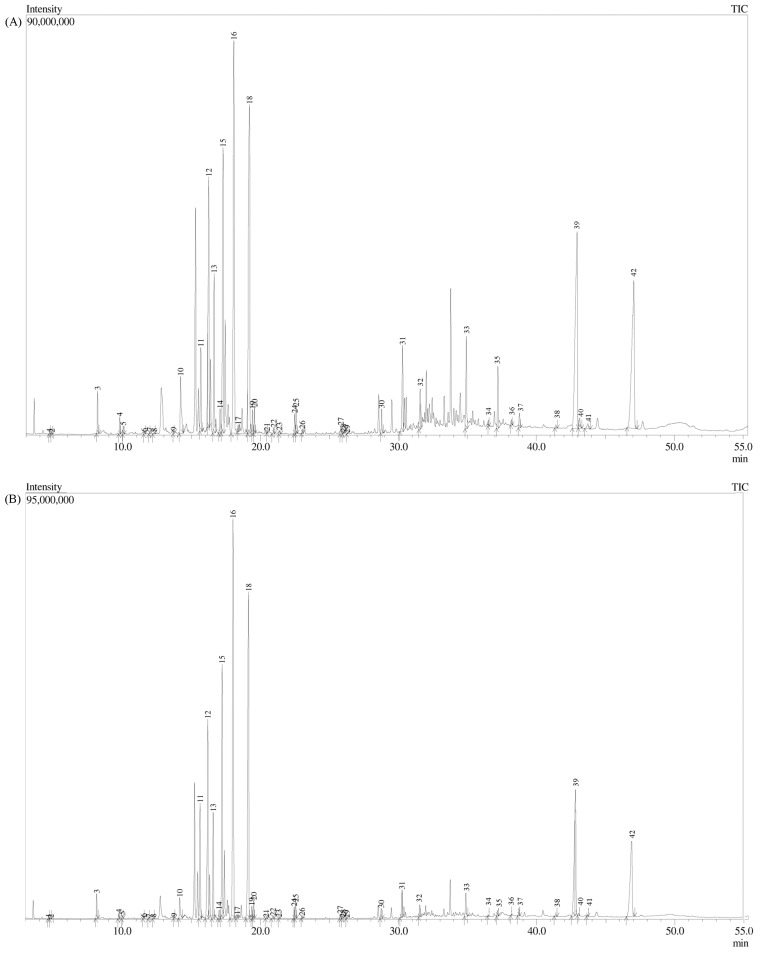
GC-MS chromatogram of (**A**) methanol extract from cv. *Lastovka* and (**B**) methanol extract from cv. *Oblica* extract after derivatization with trimethylsilyl (TMS). Peak assignment is given in Table S2.

To further explore the phytochemical profile and expand the knowledge of metabolites from olive vegetative buds, we have evaporated methanol and performed trimethylsilyl (TMS) derivatization of extracts. By doing so, 42 compounds have been identified, most of which belong to the chemical class of sugars (13 compounds), followed by phenolic (6 compounds), fatty acids (5 compounds), and triterpenoid acids (4 compounds) (Figure 1, Table S2).

Along with the chemical characterization of olive vegetative buds, their biological properties were screened as well. Both EOs and extracts of cvs. *Lastovka* and *Oblica* were subjected to antioxidant, antimicrobial, and cytotoxic analyses.

Antioxidant potential was evaluated with two different methods, ORAC and DPPH. Both methods showed superior antioxidant potential for bud extracts in comparison to the EOs. From the data in Table 3, it can also be concluded that all cv. *Lastovka* isolates have higher antioxidative potential than cv. *Oblica*, measured by both methods.

To test antimicrobial activity against foodborne pathogens, the minimal inhibitory concentration (MIC) and minimal bactericidal concentration (MBC) were determined for EOs and for extracts on Gram-positive and Gram-negative bacteria (Table 4). The tested EOs did not show antimicrobial activity or inhibited bacterial growth at a concentration of 4 mg/mL. On the other hand, the bud extracts effectively inhibited the bacterial growth of almost all tested bacteria at a concentration of 4 mg/mL. Moreover, an MIC of 2 mg/mL against *L. monocytogenes* was observed for both extracts.

**Figure 2:**
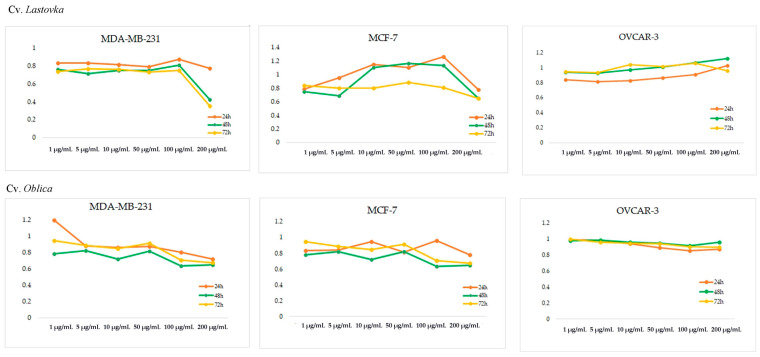
Percentage of metabolically active human breast adenocarcinoma (MDA-MB-231), human breast metastatic adenocarcinoma (MCF7), and human ovarian carcinoma (OVCAR-3) cell lines after 24 h, 48 h, and 72 h of incubation with different concentrations of olive bud extract from cvs. *Lastovka* and *Oblica*.

The cytotoxic activity of olive bud EOs and extracts from cvs. *Lastovka* and *Oblica* were tested against three human carcinoma cell lines: breast adenocarcinoma (MDA-MB-231), breast metastatic adenocarcinoma (MCF-7), and ovarian carcinoma (OVCAR-3). The results of the percentage of metabolically active cells and *p*-values for tested concentrations after 24 h, 48 h, and 72 h of incubation are shown in Figure 2, Figure S3, and Table S3. Generally, EOs showed very weak activity, and none of the samples reached the IC_50_ value regardless of incubation time or cell line tested. Methanol extracts of cv. *Lastovka* showed the best results, especially in the case of the MDA-MB-231 cell line reaching the IC_50_ value of 150 μg/mL for both 48 h and 72 h incubation times. Methanol extracts from cv. *Oblica* reached ca. 75% inhibition at concentration of 200 μg/mL after 72 h for the same cell line. Additionally, both cv. methanol extracts exhibited the same 75% inhibition at 200 μg/mL activity for the MCF-7 cancer cell line as well. No IC_50_ was reached for the OVCAR-3 cell line.

## 4. Discussion

In recent years, interest in natural products and herbal medicine has greatly increased, but the embryonic parts of plants are still largely unexplored. Phytochemicals from olive vegetative buds were scarcely investigated. This is the first report of volatile compounds from olive bud EOs as well as their biological activities. Essential oils are widely used for different applications because of their antibacterial, antifungal, and insecticidal properties, most often in perfumery, in makeup and sanitary products, as food preservers and additives, in agriculture, in pharmacy as natural remedies, and in dentistry [40].

A great diversity of volatile compounds from olive vegetative bud EOs was found in Croatian cvs. *Lastovka* and *Oblica*. As already mentioned, we identified 108 volatile compounds from cv. *Lastovka* and 110 compounds from cv. *Oblica*; altogether, 113 different volatile compounds from 18 different compound classes were found in EOs from olive vegetative buds (Table S1, Figure S1). Hydrocarbons (aliphatic and aromatic) were the most abundant class of molecules in both EOs. Saturated and unsaturated hydrocarbons often serve as the key signal for chemical mimicry, acting as female mating signals and attracting male insects, which makes them an important part of the pollination mechanism [41,42]. One of the most abundant compounds with the largest differences between cultivars was 1,3,5-trimethoxybenzene, an anisole derivative derived from phenylpropanoid metabolic pathways, with a yield of 12.60% in cv. *Lastovka* and 2.12% cv. *Oblica*. Until now, it was mostly found in rose floral scent (family Rosaceae) [43] but also found in Eugenia confuse leaf EO from the Myrtaceae family [44]. Other abundant classes of molecules (in amount > 2%) are aldehydes, alcohols, ketones, fatty acids, and monoterpene alcohols. Aldehydes and ketones are associated with sweet and sometimes pungent odors and have many other biological properties. Aldehydes in EOs are often related to antibacterial properties as well as immunomodulatory properties [45], while ketones from EOs should be used with great caution since they can have neurotoxic effects [46]. Monoterpene alcohols have similar characteristics as aldehydes but are generally more potent compounds and can also act as insecticides and repellents against pests [47].

Jurešić Grubešić et al. [48] studied volatile compounds present in olive leaf EO of cv. *Oblica* during a 6-month period (from December to May). A comparison of EOs from buds and leaves both sampled in April did not result in a great degree of similarity in volatile profiles. The most represented class of compounds sampled in April in leaf EO was ketones: *β*-ionone (20.48%), α-ionone (18.56%), and (*E*)-*β*-damascenone (5.02%). In our study, *β*-ionone (0.43%) and *β*-damascenone (0.1%) were found in much smaller amounts in cvs. *Oblica* and *Lastovka* (0.29% and 0.04%, respectively). Popović et al. [28] investigated the volatiles of olive leaf EO from cvs. *Oblica* and *Lastovka* during a three-month period (from August to October), also stating that the group of the most abundant compounds in all months was ketones, namely dihydrodehydro-*β*-ionone for cv. *Oblica* (22.53%) and (*E*)-*β*-damascenone (15%) for cv. *Lastovka*, followed by the class of sesquiterpenes and, only afterward, the class of aldehydes.

Studies of phytochemicals from bud extract are scarce and are mostly performed on particular phenolic compounds [49,50]. Phenolic compounds have a role during fruit development, with OLE being one of the most abundant compounds in olive fruit and also in the bud. Malik et al. [26] studied the OLE level in the transition from vegetative bud to mature black fruit and revealed that the highest OLE concentrations were in the vegetative bud stage, and the results were later confirmed by Taamalli et al. [27]. Our results confirm high OLE levels in vegetative bud extract; however, the compound with the highest concentration in buds for cv. *Lastovka* was oleacein. When comparing OLE levels in our study (2.05 and 2.69 mg/g fresh weight, FW, for cv. *Lastovka* and *Oblica*, respectively) with those of Malik et al. [26] (with 58.36 mg/g FW), it may be concluded that the difference in content could come from sample preparation before storage, since we did not freeze buds in liquid nitrogen prior to storage at −80°C. Cecchi et al. [51] also showed that the absence of liquid nitrogen treatment prior to unripe olive fruit storage results in a loss of OLE as much as 68%, but since the matrixes of the olive bud and fruit are different, a similar study should be performed for olive buds. The main category of olive fruit phenols are secoiridoids, including oleacein (3,4-DHPEA-EDA), which also represents one of the most abundant phenol compounds of extra virgin olive oil. In addition to oleacein, other secoiridoids such as oleuropein aglycone (3,4-DHPEA-EA) and oleocanthal (*p*-HPEA-EDA) are also present in large concentrations [52,53]. Other phenolic compounds found in higher amounts were phenolic alcohol 3-hydroxytyrosol, terpene glycoside oleuroside, glycosylated flavonoid rutin, and phenylpropanoid glycoside verbascoside. Phenolic compounds derived from olive fruit, leaves, and oil are largely responsible for their beneficial health effects [54].

Furthermore, we derivatized bud extract and performed GC-MS analysis to broaden the knowledge and the spectrum of existing compounds (Figure 1, Table S2). Dastkar et al. studied the differential expression of genes in buds of ON- vs. OFF-crop trees and found differences in the expression of genes related to carbohydrate metabolism as well as in genes involved in the secondary metabolism pathway—precisely, genes involved in phenolic biosynthesis [55]. Most of the identified compounds belong to the class of sugars (13 from 42 identified). Several phenolic compounds have also been identified, followed by fatty acids, triterpenoid acids, and organic acids. Fatty acids from olive fruit and EVOO, especially monounsaturated fatty acids (MUFAs), are widely known for their health benefits, especially on the cardiovascular system. Oleic acid is a ω-9 fatty acid, one of the most abundant MUFAs in EVOO, and is often associated with beneficial anti-inflammatory effects and improvement of immune system function [56]. Linoleic acid is a ω-6 essential fatty acid and cannot be synthesized in humans. There are a lot of controversies regarding the health implications of linoleic acid, but if consumed moderately and mostly used as a replacement for solid fats, it could be beneficial for the improvement of cardiovascular risk as well as long-term glycemic control and insulin resistance [57,58]. Pentacyclic triterpenes (including triterpenoid acids and alcohols) from olive extracts were previously studied and showed various biological benefits, such as immunomodulatory, anti-inflammatory, antioxidant, anticancer, antiviral, and antimicrobial activity [59].

As indicated by the results obtained using DPPH and ORAC assays (Table 3), all tested extracts exhibited antioxidant activity. By comparing the results obtained from both methods, the best ability to neutralize free radicals was shown by extracts of cv. *Lastovka*, both for EOs and phenolics. However, as expected, phenolic extracts of both cultivars yielded better results than bud EO extracts. The DPPH IC_50_ value for cv. *Lastovka* bud phenolic extracts and EO were 0.274 mg/mL and 30.51 mg/mL, respectively. ORAC values, expressed as μmol of Trolox equivalents (TE) per gram of extract, were much higher for cv. *Lastovka* phenolic bud extracts (1835.42) than EO extract (139.95). There is a lack of information about the antioxidant activity of olive buds in contrast to other olive tree parts such as olive fruits, olive leaves, and olive oil [60]. To the best of our knowledge, this is the first study that investigated the antioxidant activity of EOs and phenolic extracts from olive buds from two domestic olive cultivars, cvs. *Lastovka* and *Oblica*, using two methods, DPPH and ORAC.

Rekik et al. [60] measured the antioxidant activity of olive flower extracts using a DPPH radical scavenging assay, which ranged from 5.24 to 11.37 µg/mL, and concluded that the antioxidant activity increases with the development stage of the flower. Kouka et al. [61] tested four olive blossom polyphenolic extracts using the same method and obtained IC_50_ values from 40.5 to 73.25 µg/mL. Their results can be related to a certain extent to the results from our study, where the obtained values were weaker, 274 µg/mL for cv. *Lastovka* and 385 µg/mL for cv. *Oblica*. It is reported that hydroxytyrosol, due to its structure, has beneficial antioxidant, antimicrobial, anti-inflammatory, and anticancer properties [62]. The higher concentration of 3-hydroxytyrosol in cv. *Lastovka* extracts could be responsible for the better antioxidant activity compared to cv. *Oblica* extracts. Jurišić Grubešić et al. [48] studied the antioxidant capacity of EOs of cv. *Oblica* leaves using DPPH and ORAC methods, with values for the DPPH method ranging from 23.58 to 130.71 mg/mL and for ORAC from 4.43 to 73.12 µmol TE. When compared to our results of DPPH measurements, IC_50_ (cv. *Lastovka* 30.51 and cv. *Oblica* 55.36 µmol TE) results were in favor of olive bud EO, that is, bud EO showed a better antioxidant capacity than leaf EO. The same results were obtained using the ORAC method, where only one of six measurements (conducted in February; 73.12 µmol TE) gave a better result compared to the results obtained for cv. *Oblica* (43.11 µmol TE). The ORAC method was used for the assessment of monitoring quenching free peroxyl radicals. Šimat et al. [63] tested the antioxidant activity of olive leaf extracts from six Mediterranean olive cultivars using the ORAC method, among others, and the antioxidant activity was in favor of cv. *Oblica* leaf extracts, since the reported values were higher for *Oblica* than the *Lastovka* cultivar.

The antioxidant activity of parts of the olive tree is related to different groups of bioactive components such as fatty acids, triterpenic acids, polyphenols, and phytosterols as well as their synergistic effect, since it is not possible to predict the total antioxidant potential of the samples from the antioxidant activity of individual compounds [64,65]. Our results showed a higher presence of compounds such as tripenoids, acids and alcohols (betulinic acid, ursolic acid, maslinic acid, and erythrodiol), secoiridoids (oleacein, oleuropein, and ligstroside), *β*-sitosterol, and 3-hydroxytyrosol in the extracts of cv. *Lastovka*, which could explain the better antioxidant activity in both phenolic extract and EO when compared to cv. *Oblica*. Bud extracts are overall more abundant with compounds that possess higher antioxidant properties than Eos, which was confirmed by the results of ORAC and DPPH. Bud EOs are the richest in aliphatic hydrocarbons that do not possess such activities. The total antioxidant potential of the extract is mostly due to their combined effects (synergistic, antagonistic, and additive) and not only from the antioxidant activity of individual compounds [65].

Foodborne pathogens can have a great impact on human health and cause a large number of diseases [66]. We tested the antimicrobial activity of EOs and extracts against selected foodborne pathogens, Gram-positive and Gram-negative bacteria, and the results were expressed as minimal inhibitory concentration (MIC) and minimal bactericidal concentration (MBC) (Table 4). At the highest concentration of EOs (4 mg/mL), the MIC was not recorded for any of the tested bacteria, so the MBC was not tested. The bud extracts showed much better results. The MIC was determined for all bacterial strains, and the lowest MIC (2 mg/mL) was for *L. monocytogenes*. As far as the authors know, there are no previous data for the antimicrobial activity of olive bud extracts except for several reports where the antimicrobial activity of extracts from table olives, olive leaves, olive oils, and olive mill wastewater was tested. Guo et al. [67] tested olive oil polyphenol extract (OOPE) on *L. monocytogenes* and showed that the bacterial colony did not grow at an OOPE concentration of 1.25 mg/mL. Olive oil polyphenol extract affected the intracellular adenosine 5′-triphosphate (ATP) concentration level and cell membrane potential and led to a reduction in bacterial protein and DNA levels and a change in cell morphology. The extract from the cv. *Lastovka* variety showed slightly better results for *S. enteridis*, with an equal MIC and MBC concentration of 4 mg/mL, whereas for cv. *Oblica,* the MIC was reached at 4 mg/mL but the MBC could not be determined. A previous study by Liu et al. [68] on the antimicrobial activity of olive leaf extract (OLE) on *S. enteridis*, *L. monocytogenes,* and *E. coli* showed that, at a concentration of 62.5 mg/mL, the growth of *S. enteritidis* and *L. monocytogenes* was completely inhibited. The antimicrobial activity against the Gram-positive bacteria *E. faecalis* was also tested for the bud extracts of cultivars, and the MIC as well as the MBC was obtained at a concentration of 4 mg/mL. A previous study on olive leaf extract against *E. faecalis* inhibited bacterial growth at a concentration of 0.60 mg/mL, while the MBC could not be determined [69]. In the same study, an extract of table olives was also tested against *E. faecalis*, and the MIC was achieved at a concentration of 5 mg/mL, while the MBC could not be determined. The growth of *S. aureurs*, *B. cereus,* and *E. coli* was also affected by olive bud extracts of both cultivars, with MIC values of 4 mg/mL and the same MBC values for *E.coli* and *S. aureurs* (only for cv. *Oblica* extract). Šimat et al. [63] studied the antibacterial activity of olive leaf extract against the same bacterial strains. Olive leaf extract showed inhibitory activity against *S. aureurs* (MIC and MBC = 2 mg/mL) and *B. cereus* (MIC and MBC = 4 mg/mL for cv. *Lastovka*; MIC and MBC = 8 mg/mL for cv. *Oblica*), while no antimicrobial activity was detected against *E. coli*.

Phenolic compounds are a large and diverse class of compounds with different effects on microorganisms. Their structure is related to antibacterial activity, which can be mediated by different mechanisms [70]. In addition to individual phenolic compounds, a group of phenols can also interact and have a synergistic, additive, or antagonistic effect [36]. Other than phenolics, triterpenoid acids also act against different microorganisms [71]. The extract of industrial olive oil waste is rich in oleanolic and maslinic acid. Blanco-Cabra et al. [72] tested both compounds along with their derivatives against several bacterial strains including *S. aureus*, *E. faecalis*, and *E. coli*. The MIC was determined for maslinic acid in very low concentrations for *S. aureus* and *E. faecalis* (15 µg/mL for both strains), while there was no activity for *E. coli*. Similar results were obtained for oleanolic acid, but MIC values were slightly higher for *S. aureus* and *E. faecalis* (30 µg/mL for both strains), and there was no activity for *E. coli*. Oleanolic and maslinic acid derivatives showed even better results on tested bacterial strains and great antibiofilm activity for *S. aureus*. Overall, the chemical profile correlates with antimicrobial activity; extracts rich in phenolics and triterpenoid acids had better results than EOs that were high in hydrocarbons.

The Mediterranean diet is known to be linked with lower incidences of major illnesses such as cancer and cardiovascular disease. Frequent consumption of olives and olive oil, rich in antioxidant compounds that possess chemoprotective effects, is one of the different nutritional habits of the population from the Mediterranean basin [73]. Several authors have studied the effect of olive, EVOO, and olive leaf extract on different cancer cell lines, but there are no studies performed on extracts from olive bud. EOs and extracts of cvs. *Lastovka* and *Oblica* were tested for cytotoxic activity against human breast adenocarcinoma MDA-MB-231, human breast metastatic adenocarcinoma MCF7, and human ovarian carcinoma OVCAR-3 cell lines (Figure 2 and Figure S3 and Table S3). Essential oil had no activity for any of the tested cell lines (Figure S3).

Methanol extracts showed better results than EOs for both MDA-MB-231 and MCF-7 cell lines. The OVCAR-3 cell line was equally resistant to both isolates from both cultivars for the tested concentrations. Methanol extracts of cv. *Lastovka* showed somewhat better results, especially for the MDA-MB-231 cell line, where IC_50_ was reached after 48 and 72 h of incubation (150 μg/mL for both measurements). Methanol extracts from cv. *Oblica* were less potent but reached 75% inhibition at a concentration of 200 μg/mL after 72 h for the same cell line as well as for the MCF-7 cell line.

Benot-Dominguez et al. [74] studied the effect of olive leaf extract on different cancer cell lines, including MDA-MB-231 and OVCAR-3, as well as on nontumoral cells and found that OLE specifically inhibits MDA-MB-231 and OVCAR-3 cell viability (IC_50_ = 200 μg/mL) without affecting nontumoral cells. Oleuropein was a major component of OLE extracts (87% of the total components) and was able to induce a cytotoxic effect on both cell lines. The results show that OLE has multiple effects on cancer cell lines: it promotes cell cycle arrest, promotes apoptosis, selectively increases ROS production and alters the protein levels of oxidative stress pathway-related proteins, and compromises mitochondrial function. Elamin et al. [75] studied the cytotoxic effects of OLE, a major phenol from olive oil, on human breast adenocarcinoma MDA-MB-231 and human breast metastatic adenocarcinoma MCF7 cell lines. The authors determined specific cytotoxicity against breast cancer cells, for MDA-MB-231 with LC_50_ = 200 µM and for MCF7 with LC_50_ = 150 µM. Other than OLE, cytotoxic effects on MCF-7 and MDA-MB-231 cell lines were also found significant for other phenolic compounds, such as verbascoside [76] and hydroxytyrosol [77,78]. Pentacyclic triterpenes are also potent cytotoxic molecules and are known to have a cytotoxic effect on all the cancer cell lines used in this study [79].

Overall, the results for the tested biological activities of olive buds show a moderate but promising result. Since phenolics from other olive extracts seem to have the largest biological effect on metabolic disorders and the cardiovascular system, further research can be focused in that direction to examine whether this combination of phenolics and triterpenes could result in a similar or better effect. Since gemmotherapy is a scarcely investigated area, these results point to the potential hidden in embryological tissues and the need for a comprehensive search in this area for novel, promising natural compounds and/or their synergistic effect.

## 5. Conclusions

Regardless of the fact that *Olea europaea* L. is one of the world’s most important and studied crops originating from the Mediterranean basin, phytochemicals from olive vegetative buds are scarcely investigated at present. A comprehensive chemical analysis of olive vegetative bud from two Croatian cultivars revealed numerous compounds in EO and methanol extract that could be of great use for nutraceutical or biotechnological application.

Olive buds are rich in phenolics, especially oleacin, oleuropein, and hydroxytyrosol. Previous studies on olive leaf extracts, which are widely used in phytotherapy for the treatment of various conditions such as lipid regulation, hypertension, and cardiac system protection, point to oleuropein and hydroxytyrosol as the main constituents. The results of preformed biological activities (antioxidant activity, antimicrobial activity, and cytotoxic activity) show moderate biological potential of olive bud extract and the need for further investigation in different biological systems.

Research on embryonic plant parts could lead to the discovery of novel compounds as well as their nutraceutical or biotechnological application potential. Since the entire field is understudied, a further increase in research in this area is needed.

## Figures and Tables

**Table 1:** Composition of volatiles identified in essential oils of olive vegetative buds from cvs. *Lastovka* and *Oblica*.

RI	Literature RI	Compound	*Lastovka*	*Oblica*
867	865	(*Z*)-3-Hexen-1-ol *	0.67 ± 0.14	1.03 ± 0.14
991	992	2-Pentylfuran	0.37 ± 0.01	0.65 ± 0.13
1040	1040	Benzeneacetaldehyde	2.91 ± 0.24	3.91 ± 1.04
1099	1098	Linalool *	0.17 ± 0.06	0.84 ± 0.06
1104	1105	Nonanal *	4.29 ± 0.11	4.73 ± 0.87
1111	1111	Phenylethyl Alcohol *	0.29 ± 0.02	1.30 ± 0.05
1171	1171	1-Nonanol	0.29 ± 0.03	0.84 ± 0.19
1205	1206	Decanal *	0.36 ± 0.02	0.58 ± 0.30
1254	1254	Geraniol	0.40 ± 0.02	1.10 ± 0.18
1260	1260	(*E*)-2-Decenal	0.71 ± 0.04	0.55 ± 0.09
1285	1285	Dihydroedulan II	0.31 ± 0.02	0.61 ± 0.12
1295	1298	Theaspirane A	0.28 ± 0.02	0.54 ± 0.10
1313	1315	Theaspirane B	0.35 ± 0.02	0.66 ± 0.10
1314	1316	(*E,E*)-2,4-Decadienal	0.71 ± 0.06	0.53 ± 0.05
1349	-	Methyl 5-vinylnicotinate	0.43 ± 0.06	1.49 ± 0.60
1355	1356	Eugenol *	0.60 ± 0.04	0.32 ± 0.02
1400	1400	Tetradecane	0.85 ± 0.07	0.88 ± 0.14
1407	1405	1,3,5-Trimethoxybenzene *	12.60 ± 0.97	2.12 ± 0.30
1463	1465	2,6,10-Trimethyltridecane	0.54 ± 0.07	0.81 ± 0.07
1512	1512	Butylated hydroxytoluene	0.49 ± 0.12	0.53 ± 0.40
1600	1600	Hexadecane	0.59 ± 0.02	0.65 ± 0.07
1651	1656	Neointermedeol	n.d.	0.55 ± 0.39
1670	1669	(*E,E*)-6,8-Heptadecadiene	0.88 ± 0.10	0.55 ± 0.17
1677	1692	(*Z*)-3-Heptadecene	16.25 ± 1.60	8.13 ± 2.15
1700	1700	Heptadecane	2.57 ± 0.21	1.37 ± 0.35
1845	1845	Hexahydrofarnesyl acetone	1.02 ± 0.14	2.54 ± 0.66
1880	1904	Homosalate	0.17 ± 0.01	0.53 ± 0.1
1900	1900	Nonadecane	1.50 ± 0.06	1.46 ± 0.44
1965	1965	Hexadecanoic acid	1.06 ± 0.15	2.53 ± 0.55
2085	2087	1-Henicosene	2.85 ± 0.07	1.68 ± 0.55
2100	2100	Heneicosane	7.92 ± 0.16	6.02 ± 1.39
2114	2114	Phytol	0.12 ± 0.02	1.69 ± 0.35
2200	2200	Docosane	0.83 ± 0.03	0.68 ± 0.13
2274	2274	(*Z*)-9-Tricosene	2.53 ± 0.32	2.39 ± 0.61
2300	2300	Tricosane	7.08 ± 0.55	4.02 ± 0.67
2500	2500	Pentacosane	2.04 ± 0.18	1.51 ± 0.27
2700	2700	Heptacosane	2.05 ± 0.25	4.10 ± 0.29
2800	2800	Octacosane	0.31 ± 0.03	0.76 ± 0.06
2821	2833	Squalene	0.54 ± 0.08	0.28 ± 0.03
2888	2900	Nonacosane	3.81 ± 0.44	7.93 ± 0.5
3100	3100	Hentriacontane	1.28 ± 0.14	2.10 ± 0.16

RI—Retention indices on the SH-5MS column, * coinjection with commercial standards, n.d.—not detected. Identified volatiles (in the amount > 0.5% of chromatogram peak area) are expressed as mean ± SD. Compounds were identified by mass spectra and RI comparison with NIST and Wiley libraries and commercial standards as well as with the literature [21].

**Table 2:** Phenolic compounds from olive vegetative bud extract from cvs. *Lastovka* and *Oblica*.

Phenolic Compound	*Lastovka*	*Oblica*
3-Hydroxytyrosol	43.12 ± 9.94	39.07 ± 1.71
Tyrosol	3.79 ± 0.75	3.95 ± 0.26
Caffeic acid	0.12 ± 0.05	0.20 ± 0.02
Vanilin	0.03 ± 0.01	0.59 ± 0.53
*trans*-*p*-Coumaric acid	0.13 ± 0.02	0.24 ± 0.03
Rutin	16.32 ± 2.11	20.65 ± 2.90
Verbascoside	18.38 ± 2.43	29.98 ± 2.41
Luteolin-7-glucoside	4.98 ± 0.11	8.11 ± 0.74
*trans*-*o*-Coumaric acid	1.90 ± 0.35	3.08 ± 0.38
Apigenin-7-glucoside	6.84 ± 0.84	10.82 ± 1.00
Oleacein	120.37 ± 21.18	85.11 ± 20.84
Oleuropein	59.27 ± 8.86	79.39 ± 12.67
Oleuroside	16.83 ± 2.96	15.68 ± 1.96
Ligstroside	2.58 ± 0.33	4.10 ± 0.41
Pinoresinol	3.51 ± 0.39	2.54 ± 1.05
Quercetin	8.26 ± 1.66	10.43 ± 0.22
Luteolin	1.80 ± 0.33	7.10 ± 0.92
Apigenin	2.02 ± 0.29	2.26 ± 0.04
Diosmetin	0.62 ± 0.12	0.71 ± 0.12

Results are expressed as mean ± SD. The values are represented as mg/kg of dry vegetative bud.

**Table 3:** Antioxidant potential of essential oils and extracts from the olive buds.

	Essential Oil		Extracts	
Antioxidant Assay	*Lastovka*	*Oblica*	*Lastovka*	*Oblica*
ORAC (µM TE)	139.95 ± 18.06	43.11 ± 2.53	1835.42 ± 38.31	1297.8 ± 73.82
DPPH (IC_50_, mg/mL)	30.51 ± 4.9	55.36 ± 9.6	0.274 ± 0.03	0.358 ± 0.01

µM TE—μmol of Trolox equivalents per gram of EO/extract. All results are expressed as mean ± SD.

**Table 4:** Results of the minimal inhibitory concentration (mg/mL) and minimal bactericidal concentration (mg/mL) of the olive vegetative bud essential oil and extracts against foodborne pathogens (*n* = 3).

		Gram-Positive Bacteria								Gram-Negative Bacteria			
		*S. aureus*		*B. cereus*		*L. monocytogenes*		*E. faecalis*		*E. coli*		*S. enteridis*	
		MIC	MBC	MIC	MBC	MIC	MBC	MIC	MBC	MIC	MBC	MIC	MBC
Essential oils	*Lastovka*	>4	/	>4	/	>4	/	>4	/	>4	/	>4	/
	*Oblica*	>4	/	>4	/	>4	/	>4	/	>4	/	>4	/
Extracts	*Lastovka*	4	>4	4	>4	2	4	4	4	4	4	4	4
	*Oblica*	4	4	4	>4	2	>4	4	4	4	4	4	>4

MIC—minimal inhibitory concentration; MBC—minimal bactericidal concentration.

## Data Availability

All data are included within the article and in Supplementary Material.

## Supplementary Materials

The following supporting information can be downloaded at: https://www.mdpi.com/article/10.3390/metabo13020237/s1, Table S1: Composition of volatiles identified in essential oils from olive vegetative buds of cvs. *Lastovka* and *Oblica*; Table S2: The MS spectra of corresponding trimethylsilyl (TMS) metabolites from olive vegetative bud extracts; Table S3: Significance level (*p* < 0.05) of metabolically active human breast adenocarcinoma MDA-MB-231, human breast metastatic adenocarcinoma MCF7, and human ovarian carcinoma OVCAR-3 cell lines in comparison to nontreated cell line samples after 24, 48, and 72 h of incubation with different concentrations of olive vegetative bud essential oil (a) and extract (b) from cvs. *Lastovka* and *Oblica*, Figure S1: Total ion chromatogram of olive vegetative bud essential oils from (a) cv. *Lastovka* (b) cv. *Oblica*; Figure S2: HPLC chromatograms of olive vegetative bud methanol extracts for (a) cv. *Lastovka* (b) cv. *Oblica,* at 220 (black line) and 320 nm (purple line); Figure S3: Percentage of metabolically active human breast adenocarcinoma (MDA-MB-231), human breast metastatic adenocarcinoma (MCF-7), and human ovarian carcinoma (OVCAR-3) cell lines after 24, 48, and 72 h of incubation with different concentrations of olive bud essential oils from cvs. *Lastovka* and *Oblica*.
