# Supplementary material for: In the Beginning Was the Bud: Phytochemicals from Olive (Olea europaea L.) Vegetative Buds and Their Biological Properties
Source: Metabolites. 2023 Feb 5;13(2):237. doi: 10.3390/metabo13020237 (PMC9966879; doi:10.3390/metabo13020237)
Supplement: Supplementary file 1 [file metabolites-13-00237-s001.zip › metabolites-2183410-supplementary Figures.pdf]

In the Beginning was the Bud: Phytochemicals from Olive (*Olea europaea* L.) Vegetative Buds and Their Biological Properties

Marijana Popović [1,*], Franko Burčul [2], Maja Veršić Bratinčević [1], Nikolina Režić Mužinić [3], Danijela Skroza [4], Roberta Frleta Matas [5], Marija Nazlić [6], Tonka Ninčević Runjić [7], Maja Jukić Špika [1,8], Ana Bego [1], Valerija Dunkić [6] and Elda Vitanović [1]

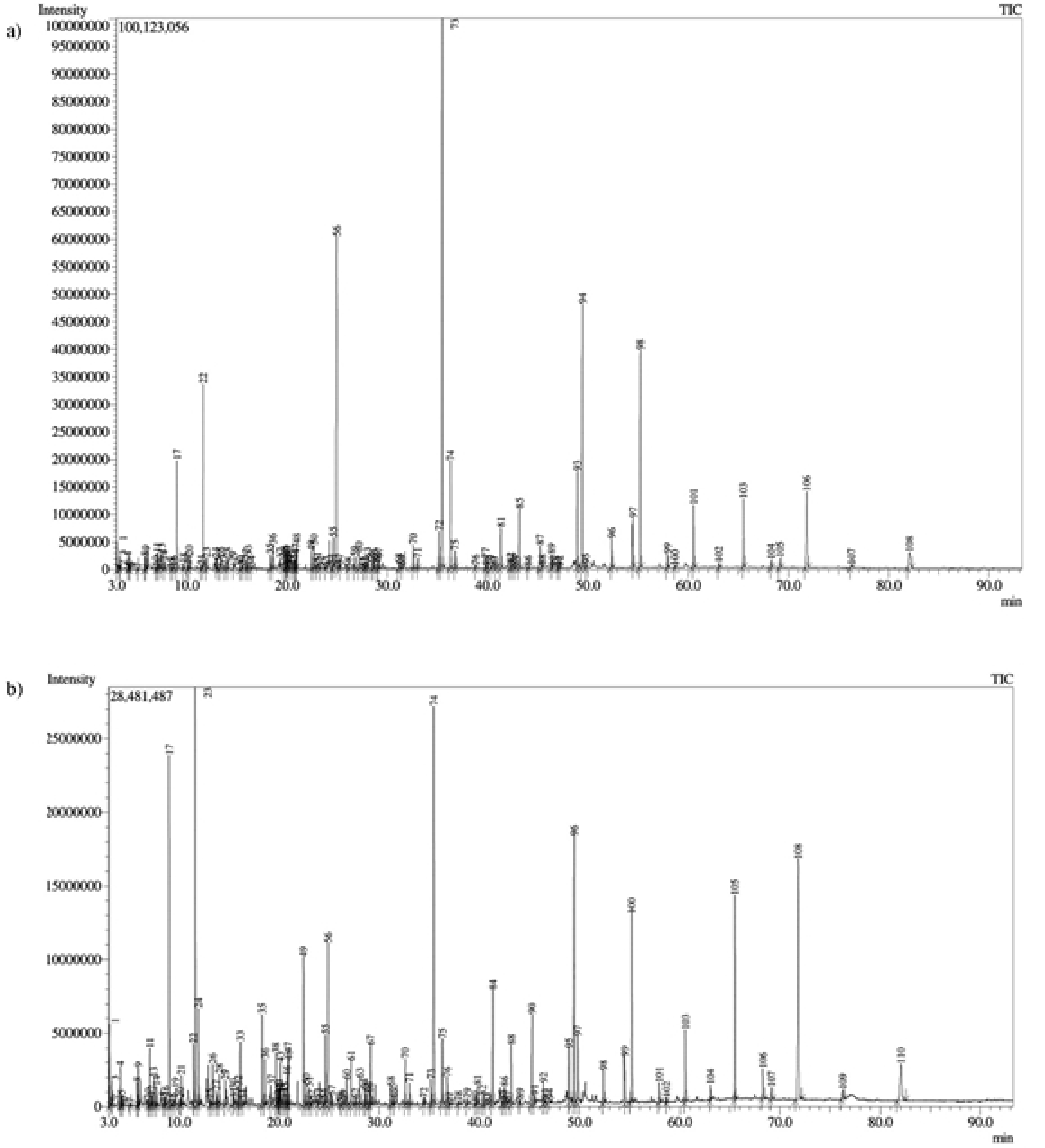

Figure S1: Total ion chromatogram of olive vegetative bud essential oils from (a) cv. *Lastovka* (b) cv. *Oblica*

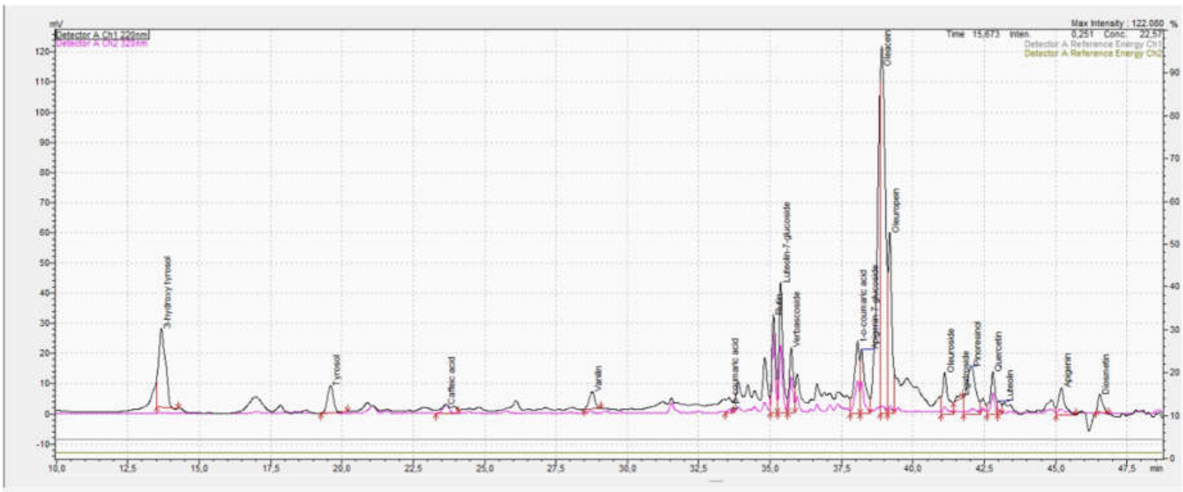

a)

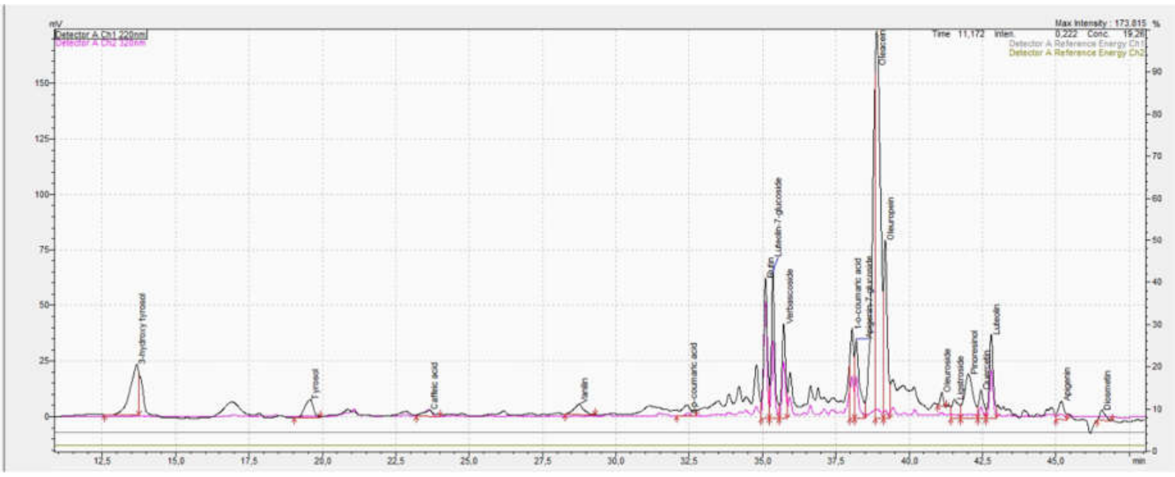

b)

Figure S2: HPLC chromatograms of olive vegetative bud methanol extracts for (a) cv. *Lastovka* (b) cv. *Oblica,* at 220 (black line) and 320 nm (purple line). Identified and quantified phenolic compounds in order of appearance: 3-hydroxytyrosol, tyrosol, caffeic acid, vanilin, *trans-p*-coumaric acid, rutin, verbascoside, luteolin-7-glucoside, *trans-o*-coumaric acid, apigenin-7-glucoside, oleacein, oleuropein, ligstroside, pinoresinol, quercetin, luteolin, apigenin and diosmetin.

Cv. *Lastovka*

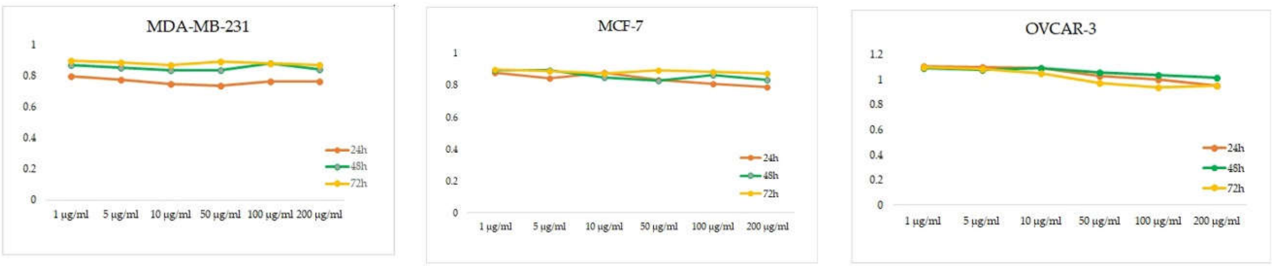

Cv. *Oblica*

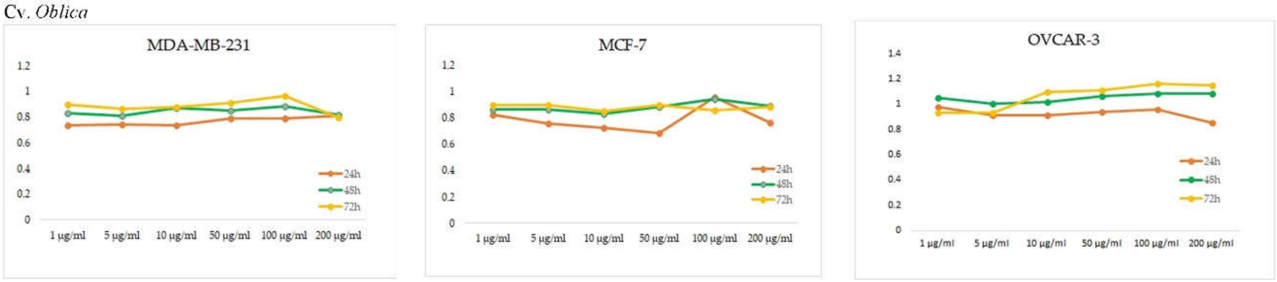

Figure S3: Percentage of metabolically active human breast adenocarcinoma (MDA-MB-231), human breast metastatic adenocarcinoma (MCF-7), and human ovarian carcinoma (OVCAR-3) cell lines after 24, 48 and 72 h of incubation with different concentrations of olive bud essential oils from cvs. *Lastovka* and *Oblica*
