# Supplementary material for: In the Beginning Was the Bud: Phytochemicals from Olive (Olea europaea L.) Vegetative Buds and Their Biological Properties
Source: Metabolites. 2023 Feb 5;13(2):237. doi: 10.3390/metabo13020237 (PMC9966879; doi:10.3390/metabo13020237)
Supplement: Supplementary file 1 [file metabolites-13-00237-s001.zip › metabolites-2183410-supplementary Tables.pdf]

In the Beginning was the Bud: Phytochemicals from Olive (*Olea europaea* L.) Vegetative Buds and Their Biological Properties

Marijana Popović [1,*], Franko Burčul [2], Maja Veršić Bratinčević [1], Nikolina Režić Mužinić [3], Danijela Skroza [4], Roberta Frleta Matas [5], Marija Nazlić [6], Tonka Ninčević Runjić [7], Maja Jukić Špika [1,8], Ana Bego [1], Valerija Dunkić [6] and Elda Vitanović [1]

Table S1: Composition of volatiles identified in essential oils of olive vegetative buds from cvs. *Lastovka* and *Oblica*

			Lastovka (%)		*Oblica* (%)	
**RI**	**Lit. RI**	**Compound**	**mean**	**SD**	**mean**	**SD**
**867**	865	(*Z*)-3-Hexen-1-ol*	0.67	0.14	1.03	0.14
**874**	875	(*Z*)-2-Hexen-1-ol	0.04	0.00	0.02	0.00
**875**	876	1-Hexanol	0.11	0.07	0.20	0.01
**901**	901	Heptanal	0.24	0.01	0.37	0.08
**904**	906	Methional	0.01	0.00	0.13	0.00
**908**	910	2,4-Hexadienal*	0.04	0.01	0.04	0.00
**931**	934	2,7-Dimethyl-oxepine	0.02	0.00	0.02	0.00
**952**	954	(*E*)-2-Heptenal	0.18	0.13	0.26	0.04
**956**	961	Benzaldehyde	0.39	0.03	0.49	0.04
**986**	987	6-Methyl-5-hepten-2-one	0.06	0.00	0.09	0.01
**991**	992	2-Pentylfuran	0.37	0.01	0.65	0.13
**996**	998	(*E,Z*)-2,4-Heptadienal	0.11	0.01	0.13	0.02
**1002**	1001	Octanal	0.29	0.01	0.33	0.08
**1009**	1008	(*E,E*)-2,4-Heptadienal	0.20	0.02	0.22	0.03
**1026**	1025	Limonene	0.02	0.00	0.03	0.00
**1031**	1033	Benzyl alcohol	0.06	0.00	0.14	0.01
**1040**	1040	Benzeneacetaldehyde	2.91	0.24	3.91	1.04
**1047**	1011	3-Carene*	n.d.	-	0.02	0.00
**1056**	1056	(*E*)-2-Octenal	0.13	0.00	0.19	0.04
**1067**	1067	(*E*)-2-Octen-1-ol	0.09	0.01	0.07	0.02
**1070**	1068	1-Octanol	0.35	0.03	0.47	0.06
**1099**	1098	Linalool*	0.17	0.06	0.84	0.06
**1104**	1105	Nonanal*	4.29	0.11	4.73	0.87
**1111**	1111	Phenylethyl Alcohol*	0.29	0.02	1.30	0.05
**1137**	1138	Methyl nicotinate	0.03	0.01	0.08	0.01
**1143**	1154	6-Methyl-1-octanol	0.27	0.02	0.50	0.07
**1152**	1151	(*E,Z*)-2,6-Nonadienal	0.07	0.00	0.18	0.02
**1158**	1159	(*E*)-2-Nonenal	0.25	0.01	0.39	0.06
**1171**	1171	1-Nonanol	0.29	0.03	0.84	0.19

**1189**	1188	α-Terpineol	0.06	0.00	0.19	0.03
**1191**	1191	Methyl salicylate*	0.11	0.02	0.04	0.01
**1200**	1200	Dodecane	0.23	0.03	0.24	0.04
**1205**	1206	Decanal*	0.36	0.02	0.58	0.30
**1213**	1214	(*E*,*E*)-2,4-Nonadienal	0.03	0.00	0.02	0.01
**1254**	1254	Geraniol	0.40	0.02	1.10	0.18
**1260**	1260	(*E*)-2-Decenal	0.71	0.04	0.55	0.09
**1275**	1276	Nonanoic acid	0.11	0.06	0.37	0.05
**1285**	1285	Dihydroedulan II	0.31	0.02	0.61	0.12
**1289**	1289	Indole	0.04	0.00	0.04	0.01
**1291**	1292	Dihydroedulan IA	0.27	0.02	0.06	0.01
**1292**	1293	(*E*,*Z*)-2,4-Decadienal	0.22	0.02	0.11	0.01
**1294**	1294	2-Undecanone	0.01	0.00	0.06	0.00
**1295**	1298	Theaspirane A	0.28	0.02	0.54	0.10
**1300**	1300	Tridecane	0.05	0.01	0.11	0.01
**1307**	1307	Undecanal	0.09	0.01	0.15	0.03
**1311**	1312	2-Vinylguaicol	0.18	0.02	0.38	0.04
**1313**	1315	Theaspirane B	0.35	0.02	0.66	0.10
**1314**	1316	(*E*,*E*)-2,4-Decadienal	0.71	0.06	0.53	0.05
**1349**	-	Methyl 5-vinylnicotinate	0.43	0.06	1.49	0.60
**1355**	1356	Eugenol*	0.60	0.04	0.32	0.02
**1363**	1363	2-Undecenal	0.14	0.01	0.24	0.04
**1369**	1370	n-Decanoic acid	0.02	0.01	0.10	0.05
**1383**	1384	β-Damascenone	0.04	0.00	0.10	0.01
**1397**	1397	(*Z*)-Jasmone	0.08	0.00	0.09	0.01
**1400**	1400	Tetradecane	0.85	0.07	0.88	0.14
**1407**	1405	1,3,5-Trimethoxybenzene*	12.60	0.97	2.12	0.30
**1416**	1416	Caryophyllene*	0.14	0.03	0.12	0.02
**1437**	1436	Dihydro-β-ionone	0.01	0.00	0.04	0.01
**1444**	1449.2	Dihydro-β-ionol	n.d.	-	0.08	0.00
**1452**	1452	(*E*)-Geranyl acetone	0.32	0.02	0.29	0.06
**1463**	1465.1	2,6,10-Trimethyltridecane	0.54	0.07	0.81	0.07
**1474**	1473	1-Dodecanol	0.03	0.01	0.12	0.01
**1478**	1478	Germacrene D	0.03	0.00	n.d.	-
**1484**	1484	(*E*)-β-Ionone	0.29	0.02	0.43	0.04
**1496**	1496	2-Tridecanone	0.15	0.01	0.33	0.05
**1500**	1500	Pentadecane	0.22	0.01	0.23	0.04
**1508**	1508	α-Farnesene	0.18	0.00	0.21	0.03
**1512**	1512	Butylated Hydroxytoluene	0.49	0.12	0.53	0.40
**1563**	1562	Nerolidol	0.20	0.04	0.37	0.11
**1571**	1571	3-Methyl-pentadecane	0.07	0.00	0.12	0.00
**1600**	1600	Hexadecane	0.59	0.02	0.65	0.07
**1612**	1612	Tetradecanal	0.24	0.00	0.31	0.03

**1651**	1656	Neointermedeol	n.d.	-	0.55	0.39
**1670**	1669	(*E,E*)-6,8-Heptadecadiene	0.88	0.10	0.55	0.17
**1677**	1692	(*Z*)-3-Heptadecene	16.25	1.60	8.13	2.15
**1700**	1700	Heptadecane	2.57	0.21	1.37	0.35
**1714**	1714	Pentadecanal	0.48	0.02	0.50	0.04
**1722**	1721	Farnesol	n.d.	-	0.06	0.01
**1746**	1745	7-Methyl-heptadecane	n.d.	-	0.08	0.01
**1772**	1771	3-Methyl-heptadecane	0.07	0.00	0.12	0.01
**1793**	1793	1-Octadecene	n.d.	-	0.05	0.01
**1800**	1800	Octadecane	0.26	0.01	0.28	0.01
**1803**	1805	2-Ethylhexyl salicylate	0.07	0.01	n.d.	-
**1816**	1817	Hexadecanal	0.07	0.00	0.17	0.03
**1828**	1828	Isopropyl myristate	0.02	0.01	0.03	0.01
**1845**	1845	Hexahydrofarnesyl acetone	1.02	0.14	2.54	0.66
**1874**	1875	1-Nonadecene	0.15	0.01	0.19	0.08
**1880**	1904	Homosalate	0.17	0.01	0.53	0.10
**1888**	-	(*Z*)-5-Nonadecene	0.05	0.00	0.15	0.07
**1900**	1900	Nonadecane	1.50	0.06	1.46	0.44
**1927**	1927	Methyl palmitate	0.04	0.00	0.07	0.02
**1965**	1965	Hexadecanoic acid	1.06	0.15	2.53	0.55
**1972**	1972	3-Methyl-nonadecane	0.09	0.01	0.30	0.06
**2000**	2000	Eicosane	0.35	0.01	0.47	0.10
**2005**	2006	5,5-Diethylheptadecane	0.01	0.00	0.09	0.00
**2020**	2021	Octadecanal	0.02	0.00	0.11	0.03
**2027**	2032	Isopropyl palmitate	0.04	0.01	n.d.	-
**2085**	2087	1-Henicosene	2.85	0.07	1.68	0.55
**2103**	2100	Heneicosane	7.92	0.16	6.02	1.39
**2114**	2114	Phytol	0.12	0.02	1.69	0.35
**2200**	2200	Docosane	0.83	0.03	0.68	0.13
**2274**	2274	(*Z*)-9-Tricosene	2.53	0.32	2.39	0.61
**2303**	2300	Tricosane	7.08	0.55	4.02	0.67
**2400**	2400	Tetracosane	0.44	0.04	0.40	0.06
**2429**	2430	Docosanal	0.05	0.01	0.09	0.01
**2502**	2500	Pentacosane	2.04	0.18	1.51	0.27
**2600**	2600	Hexacosane	0.16	0.03	0.29	0.04
**2693**	2700	Heptacosane	2.05	0.25	4.10	0.29
**2800**	2800	Octacosane	0.31	0.03	0.76	0.06
**2821**	2833	Squalene	0.54	0.08	0.28	0.03
**2888**	2900	Nonacosane	3.81	0.44	7.93	0.50
**2999**	3000	Triacontane	0.15	0.02	0.38	0.01
**3103**	3100	Hentriacontane	1.28	0.14	2.10	0.16
		**Total chromatogram identified:**	92.08 %		88.59 %	
		Alcohols	1.69		3.23	

Aldehydes	9.69	11.29
Ketones	1.99	3.97
Esters	0.23	0.21
Fatty acids	1.19	3.00
Phenols	0.66	0.91
Anisole derivative	12.60	2.12
Monoterpens	0.02	0.05
Sesquiterpenes	0.35	0.33
Triterpens	0.54	0.28
Monoterpene alcohols	0.63	2.13
Sesquiterpene alcohol	n.d.	0.06
Diterpene alcohols	0.12	1.69
Furans	0.37	0.65
Heterocyclic compounds	1.70	3.96
Saturated hydrocarbons	33.48	35.36
Unsaturated hydrocarbons	22.71	13.14
Aromatic hydrocarbones	4.11	6.19

Table S2: The MS spectra of corresponding TMS metabolites from olive vegetative bud extracts. MS spectra were obtained from the Golm Metabolome Database[G], MassBank [M], NIST Chemistry WebBook [N], by derivatized analytical standard [S], as well as by comparison with literature data[L].

	RI	Compound	Relative Intensity (m/z)
1	1323	Glyceric acid, 3TMS derivative [G, N]	293 (13), 292 (40), 205 (14), 189 (49), 147 (61), 133 (17), 117 (11), 103 (22), 102 (18), 73 (100)
2	1332	(E)-2-Butenedioic acid, 2TMS derivative [N]	247 (9), 246 (17), 245 (100), 148 (6), 147 (39), 143 (14), 133 (6), 75 (17), 73 (46), 45 (10)
3	1499	Malic acid, 3TMS derivative [G, N]	245 (13), 233 (24), 190 (8), 189 (11), 147 (53), 133 (9), 75 (9), 74 (8), 73 (100), 45 (8)
4	1573	Tyrosol, 2TMS derivative [N]	283 (5), 282 (20), 267 (13), 193 (11), 180 (14), 179 (100), 103 (11), 75 (5), 73 (49), 45 (7)
5	1585	Threonic acid, 4TMS derivative [G,N]	293 (16), 292 (55), 220 (28), 217 (20), 205 (24), 148 (9), 147 (58), 117 (15), 103 (13), 73 (100)
6	1655	Rhamnose, 4TMS derivative [N]	217 (6), 206 (9), 205 (20), 204 (100), 191 (21), 189 (8), 147 (14), 130 (5), 74 (4), 73 (40)
7	1673	Arabinopyranose, 4TMS derivative (isomer 2) [N]	218 (16), 217 (85), 205 (14), 204 (72), 191 (61), 147 (30), 107 (11), 75 (15), 73 (100), 45 (11)
8	1689	Arabinose, 4TMS derivative [N]	219 (8), 218 (19), 217 (100), 205 (13), 204 (71), 192 (11), 191 (63), 189 (8), 147 (27), 73 (94)
9	1755	Xylitol, 5TMS derivative [G,N]	319 (24), 307 (30), 218 (27), 217 (100), 205 (19), 189 (11), 147 (34), 129 (26), 103 (48), 73 (92)
10	1780	2-(3,4-Dihydroxyphenyl) ethanol, TMS derivative [N]	371 (17), 370 (46), 269 (10), 268 (28), 267 (100), 193 (24), 179 (15), 75 (8), 73 (69), 45 (10)
11	1843	Fructofuranose (isomer 2), 5TMS derivative [N]	438 (14), 437 (35), 257 (12), 219 (10), 218 (18), 217 (92), 147 (25), 129 (8), 74 (8), 73 (100)
12	1880	Methyl-α-glucofuranoside, 4TMS derivative[N]	219 (18), 218 (51), 217 (100), 204 (22), 191 (22), 147 (19), 132 (17), 129 (46), 103 (13), 73 (77)
13	1899	Quinic acid, 5TMS derivative [G, N]	347 (16), 346 (28), 345 (100), 334 (9), 256 (9), 255 (38), 204 (10), 191 (10), 147 (26), 73 (69)
14	1922	Gluconolactone, 4TMS derivative [N]	319 (20), 229 (9), 220 (14), 217 (19), 205 (9), 204 (14), 147 (26), 129 (27), 103 (12), 73 (100)
15	1932	Glucose, 5TMS derivative [N]	218 (6), 217 (22), 206 (10), 205 (20), 204 (100), 192 (9), 191 (49), 147 (20), 129 (9), 73 (56)
16	1973	Mannitol, 6TMS derivative [G,N]	319 (34), 218 (44), 217 (52), 205 (62), 157 (34), 147 (41), 129 (15), 117 (19), 103 (27), 73 (100)
17	1990	Galactose, 5TMS derivative [N]	218 (11), 217 (45), 205 (19), 204 (83), 191 (33), 147 (22), 129 (10), 75 (18), 74 (9), 73 (100)
18	2033	Galactopyranose, 5TMS derivative (isomer 2) [N]	217 (30), 206 (12), 205 (30), 204 (100), 192 (16), 191 (78), 147 (33), 129 (12), 74 (10), 73 (94)
19	2045	β-Galactofuranose, 5TMS derivative [N]	305 (14), 219 (10), 218 (19), 217 (100), 204 (14), 191 (12), 147 (22), 129 (11), 75 (8), 73 (65)
20	2054	Palmitic Acid, TMS derivative [N]	314 (20), 313 (77), 145 (24), 132 (50), 129 (39), 117 (100), 75 (53), 73 (76), 55 (20), 43 (25)
21	2105	Ferulic acid, 2TMS derivative [N,S]	339 (24), 338 (100), 324 (19), 323 (62), 308 (51), 293 (34), 249 (39), 191 (20), 147 (30), 73(91)
22	2131	Myo-Inositol, 6TMS derivative [N]	318 (49), 306 (27), 305 (81), 265 (20), 218 (18), 217 (94), 204 (18), 191 (47), 147 (55), 73 (100)

23	2154	Caffeic acid, 3TMS derivative [G,N,S)	398 (13), 397 (28), 396 (80), 381 (19), 249 (9), 220 (17), 219 (100), 191 (15), 45 (12), 73 (61)
24	2216	Linoleic acid, TMS derivative [M, N]	337 (52), 129 (36), 117 (36), 95 (43), 81 (67), 75 (91), 73 (100), 67 (74), 55 (49), 41 (38)
25	2222	Oleic acid, TMS derivative [N]	339 (81), 145 (36), 129 (75), 117 (96), 96 (28), 75 (90), 73 (100), 55 (55), 43 (29), 41 (37)
26	2250	Stearic acid, TMS derivative [M,N]	342 (21), 341 (76), 145 (30), 132 (50), 129 (42), 117 (100), 75 (51), 73 (75), 55 (21), 43 (30)
27	2417	Oleamide, TMS derivative [N]	338 (38), 144 (62), 131 (100), 128 (43), 116 (53), 75 (71), 73 (87), 55 (29), 43 (22), 41 (26)
28	2428	Uridine, 3TMS derivative [G, M, N]	259 (26), 219 (10), 218 (28), 217 (95), 169 (12), 147 (21), 129 (13), 103 (20), 75 (10), 73 (100)
29	2448	Arachidic acid, TMS derivative [N]	369 (71), 145 (38), 132 (71), 131 (46), 129 (43), 117 (100), 75 (66), 73 (87), 55 (28), 43 (43)
30	2609	1-Monopalmitin, 2TMS derivative [N]	372 (28), 371 (100), 239 (17), 204 (19), 203 (18), 147 (31), 129 (21), 73 (61), 57 (21), 43 (21)
31	2714	Sucrose, 8TMS derivative [G,N]	437 (19), 363 (15), 362 (31), 361 (100), 271 (13), 217 (35), 169 (11)
32	2803	Glycerol monostearate, 2TMS derivative [N]	400 (29), 399 (100), 203 (17), 147 (24), 129 (17), 73 (33), 71 (12), 57 (20), 55 (12), 43 (20)
33	3055	Methyloleoside, 5TMS derivative [N]	362 (33), 361 (100), 297 (21), 217 (29), 169 (20), 165 (69), 147 (26), 129 (19), 103 (18), 73 (97)
34	3179	Chlorogenic acid, 6TMS derivative [N,S]	397 (18), 347 (17), 346 (33), 345 (99), 324 (17), 307 (44), 256 (17), 255 (67), 147 (19), 73 (100)
35	3236	Quercetin, 5TMS derivative [N,S]	651 (4), 650 (12), 649 (33), 648 (57), 647 (100), 561 (3), 560 (5), 559 (9), 73 (21), 45 (2)
36	3312	Luteolin, 4TMS derivative [N]	562 (8), 561 (27), 560 (51), 559 (100), 472 (4), 471 (10), 452 (3), 399 (6), 73 (21), 45 (3)
37	3360	β-Sitosterol, TMS derivative [G,N]	486 (44), 396 (84), 357 (70), 129 (100), 121 (34), 95 (40), 81 (34), 73 (56), 57 (40), 43 (51)
38	3569	Erythrodiol, 2TMS derivative [N]	497 (36), 496 (75), 217 (27), 216 (100), 204 (37), 203 (53), 201 (21), 189 (28), 161 (26), 73 (50)
39	3682	Oleanolic acid, 2TMS derivative [G, N]	483 (14), 482 (25), 320 (34), 204 (16), 203 (100), 202 (63), 190 (25), 189 (31), 75 (14), 73 (44)
40	3702	Betulinic acid, 2TMS derivative [N]	320 (29), 292 (26), 202 (27), 190 (44), 189 (71), 188 (26), 187 (27), 129 (27), 75 (35), 73 (100)
41	3750	Ursolic acid, 2TMS derivative [N]	321 (20), 320 (74), 203 (100), 202 (39), 190 (20), 189 (27), 133 (45), 119 (21), 75 (18), 73 (43)
42	4002	Maslinic acid, 3TMS derivative [L]	570 (22), 320 (43), 204 (20), 203 (100), 202 (69), 189 (29), 187 (20), 147 (30), 133 (20), 73 (83)

Table S3: Significance level ($p < 0.05$) of metabolically active human breast adenocarcinoma MDA-MB-231, human breast metastatic adenocarcinoma MCF7, and human ovarian carcinoma OVCAR-3 cell lines in comparison to non-treated cell line samples after 24, 48 and 72 h of incubation with different concentrations of olive vegetative bud essential oil (a) and extract (b) from cvs. *Lastovka* and *Oblica*

a)	MDA-MB-231			MCF-7			OVCAR-3		
Lastovka									
conc. (μg/mL)	24 h	48 h	72 h	24 h	48 h	72 h	24 h	48 h	72 h
1	0.095	0.157	0.099	0.282	0.049	1.000	0.649	0.515	0.338
5	0.114	0.126	0.052	0.183	0.019	0.013	0.681	0.572	0.480
10	0.147	0.084	0.074	0.308	0.010	0.024	0.705	0.525	0.708
50	0.087	0.088	0.071	0.168	0.006	0.043	0.889	0.627	0.742
100	0.098	0.170	0.053	0.107	0.050	0.004	0.996	0.755	0.457
200	0.087	0.092	0.041	0.092	0.001	0.008	0.779	0.882	0.491
Oblica									
conc. (μg/mL)	24 h	48 h	72 h	24 h	48 h	72 h	24 h	48 h	72 h
1	-	0.085	0.081	0.132	0.024	0.070	0.903	0.604	0.383
5	0.075	0.052	0.039	0.061	0.002	0.043	0.623	0.979	0.399
10	0.128	0.156	0.064	0.044	0.018	0.010	0.611	0.840	0.248
50	0.136	0.111	0.121	0.039	0.014	0.017	0.733	0.569	0.263
100	0.136	0.201	0.578	0.669	0.039	0.009	0.811	0.414	0.124
200	0.155	0.132	0.112	0.137	0.003	0.012	0.357	0.408	0.124

b)	MDA-MB-231			MCF-7			OVCAR-3		
Lastovka									
conc. (μg/mL)	24 h	48 h	72 h	24 h	48 h	72 h	24 h	48 h	72 h
1	0.048	0.008	0.014	0.020	0.046	0.013	0.364	0.607	0.535
5	0.043	0.001	0.026	0.417	0.004	0.006	0.287	0.498	0.462
10	0.035	0.004	0.012	0.080	0.251	0.003	0.303	0.770	0.738
50	0.027	0.010	0.084	0.388	0.240	0.029	0.405	0.970	0.904
100	0.224	0.011	0.027	0.073	0.154	0.033	0.566	0.612	0.362
200	0.016	0.000	0.000	0.003	0.004	0.001	0.579	0.475	0.720
Oblica									
conc. (μg/mL)	24 h	48 h	72 h	24 h	48 h	72 h	24 h	48 h	72 h
1	0.049	0.048	0.013	0.004	0072	0.492	0.957	0.881	0.943
5	0.121	0.005	0.003	0.483	0.137	0.142	0.934	0.882	0.646
10	0.153	0.008	0.004	0.827	0.018	0.011	0.762	0.702	0.561
50	0.252	0.003	0.023	0.299	0.061	0.111	0.562	0.579	0.485
100	0.055	0.001	0.000	0.135	0.002	0.003	0.421	0.417	0.304
200	0.010	0.000	0.000	0.006	0.003	0.002	0.473	0.696	0.235
